# Assessment of completeness and legibility of handwritten prescriptions in six community chain pharmacies of Asmara, Eritrea: a cross-sectional study

**DOI:** 10.1186/s12913-020-05418-9

**Published:** 2020-06-22

**Authors:** Dawit G. Weldemariam, Nebyu Daniel Amaha, Nuru Abdu, Eyasu H. Tesfamariam

**Affiliations:** 1 Pharmacy, Hazhaz Hospital, Asmara, Eritrea P.O. Box: 5427,; 2 Department of Nutrition and Dietetics, College of Health Sciences, Mekelle University, Mekelle, Ethiopia; 3 Department of Medical Sciences, Pharmacy Unit, Orotta College of Medicine and Health Sciences, Asmara, Eritrea; 4 Department of Statistics, Biostatistics and Epidemiology, Mai-Nefhi College of Sciences, Eritrean Institute of Technology, Abardae, Eritrea

**Keywords:** Completeness, Legibility, Prescription, Community chain pharmacy, Prescription error, Eritrea

## Abstract

**Background:**

Incompleteness and illegibility of prescriptions are prescription errors that account for a high proportion of medication errors that could potentially result in serious adverse effects. Thus, the objective of this study was to assess the completeness and legibility of prescriptions filled in the community chain pharmacies.

**Methods:**

An analytical and cross-sectional study was conducted in the six government owned community chain pharmacies of Asmara, Eritrea from June 3rd to 10th, 2019 using a stratified random sampling technique. A total of 385 prescriptions were analyzed for completeness and legibility by three pharmacists (two experienced and one intern pharmacist). Descriptive statistics and multinomial logistic regression were employed using IBM SPSS® (Version 22).

**Results:**

A total of 710 drugs were prescribed from the 385 prescriptions assessed. On average, a prescription was found to have 78.63% overall completeness. In the majority of the prescriptions, patient’s information such as name, age, sex, and prescriber’s identity were present. Prescribed drugs’ information such as dose, frequency and quantity and/or duration were present in 83.7, 87.7, and 95.1% respectively. Moreover, generic names were used in 83.3% of the drugs prescribed. About half (54.3%) of the prescriptions’ legibility were classified in grade four (clearly legible) and 30.6% in grade three (moderately legible). It was observed that legibility significantly increased with an increase in percentage completeness (r_s_ = 0.14, *p* = 0.006). However, as the number of drugs written in brand name increased, legibility decreased (r_s_ = − 0.193, *p* < 0.001). Similarly, as the number of drugs prescribed increased, legibility decreased (r_s_ = − 0.226, *p* < 0.006).

**Conclusion:**

Majority of the handwritten prescriptions received in the community pharmacies of Asmara are complete and clearly legible.

## Background

A prescription is a legal and valid written order from a prescriber to a dispenser [1, 2]. Prescription writing is not simply putting a drug’s name on a piece of paper, rather it is a skill that every prescriber needs to master through learning, hard work, and experience [1, 3]. According to the World Health Organization (WHO) prescription writing guideline, a prescription should contain: name, address, telephone number of prescriber; date of the prescription, generic name of the drug, strength (dose), dosage form and total amount, label (instruction and warning); patient name, address, and age; and signature or initials of prescriber. However, there is no globally accepted standard for prescriptions, and every nation has developed its own rules and regulations [1, 2, 4]. The Eritrean National prescription writing format is almost identical that of WHO. It includes the patient card number, date, name, age, sex and address of the patient, details of drugs prescribed, prescriber’s qualification, name, and signature.

Medication errors are a major health concern that prevents the right patient from receiving the right medicine at the right dose at the right time through the correct route of administration. In the United States, medication errors are estimated to affect 1.5 million patients every year [5]. Moreover, 80,000 hospital admissions in the United Kingdom [6] and for approximately 5% of hospital admissions in Spain was due to medication errors [7]. According to Fadare et al., prescription-related errors remain significantly high in North and Central Africa and Europe [8].

Medication errors can occur in any of the medication use process: prescribing, transcribing, dispensing, administering and monitoring its effect [1, 4, 9–12]. A medication prescription error occurs when prescription decision or its writing results in a reduction of treatment effectiveness or increases the risk of harm compared to the accepted practice [9, 10, 13]. It accounts for 39 to 74% of all medication errors [11]. Nearly half of the errors occur during the prescribing stage, and the main factors that lead to increased medication error are illegible and incomplete prescription orders [9–11, 14]. Prescription errors could be classified into two types, omission error (prescription writing error) and commission error (prescription decision error). Omission error involves the absence of any of the drug details, and illegible prescriptions while commission error is a prescription decision error in which any of the drug details is wrong or given to the wrong person [11, 15]. Illegible prescriptions are difficult to interpret and/or understand what the prescriber’s intention actually is [16]. A study done by Winslow et al. concluded that 20.2% of prescription orders were illegible or readable with effort [17]. To the best of our knowledge, there is no such published research study conducted in Eritrea to assess the completeness and legibility of a prescription. Thus, the objective of this study was to assess the overall completeness and legibility of handwritten prescriptions filled in the community chain pharmacies of Asmara city, Eritrea.

## Methods

### Study design and setting

An analytical and cross-sectional study, with a quantitative approach, was conducted in six government-owned community chain pharmacies of Asmara, Eritrea. Eritrea has 13 government-owned community chain pharmacies, six of which are located in Asmara, the capital city of Eritrea with around 422,309 inhabitants (in 2017). Moreover, two of the six community chain pharmacies are found inside the National Referral Hospitals: Orotta and Halibet. Patients who cannot find the prescribed drugs in the Out-patient Department (OPD) pharmacies of hospitals often seek their medicines from these six government-owned community chain pharmacies located in Asmara. This is because there is comparably high availability and affordability of drugs in these pharmacies when compared to private community pharmacies.

### Sample size determination

The sample size was computed by using the formula [18]:

$$ n\ge \frac{NZ^2 pq}{\left({d}^2\left(N-1\right)+{Z}^2 pq\right)} $$

The total sample size (n) was calculated using the following assumptions: estimated number of prescriptions filled in 7 days in the six community pharmacies (*N* = 5320), expected proportion of prescriptions which are completely legible (p) and those illegible (q) were taken as 0.5, Z statistic for 95% level of confidence (Z = 1.96), margin of error (d) of 0.05 and 5% non-response rate. Considering the above assumptions, to have a representative sample, the least required number was 382.

### Sampling design and allocation

In order to get representative samples from each community pharmacy, stratified random sampling method was utilized. The six community pharmacies were considered as strata, and samples were taken from each community pharmacy systematically. The computed sample size was proportionally allocated among the six community pharmacies (Table 1).

**Table 1:** Sample allocation of the prescriptions

Pharmacy	Estimated number of prescriptions filled in 7 days	Samples taken
Chain Pharmacy No. 1	1479	107
Chain Pharmacy No. 2	373	27
Chain Pharmacy No. 3	511	37
Chain Pharmacy No. 4	968	70
Chain PharmacyNo. 5	1105	80
Chain Pharmacy No. 6	884	64
Total	5320	385

### Data collection tool and technique

Data was collected from prescriptions between June 3rd and 10th, 2019 using a data recording checklist form. It was designed by the researchers in such a way to enable easily retrieval and recording of information about patients, prescribers, and drugs prescribed from the patient’s prescriptions [see additional file 1]. In Eritrea, the term “prescriber” refers to medical doctors, nurses, registered nurses, health assistants, and other lower health cadres.

A prescription was assessed for its completeness based on WHO guidelines of good prescribing practice [4] and the national prescription writing guidelines. The legibility of a prescription was assessed by three pharmacists.

### Variable measurement

Omission error, a prescription writing error, was measured in percentage completeness of the patient’s details, prescriber’s identity and drug’s information, and the degree of illegibility of the prescription. Percentage completeness of prescription was calculated based on percent score. First, a score of (1) for presence and (0) for absence was assigned to the total 13 elements (*N* = 13) of the patient’s information, prescriber’s identity, medication information and other information (presence of Date). Then, the assigned scores were summated and divided by the total number of elements (13 for prescriptions with one drug, 18 for prescriptions with two drugs and so on) and multiplied by 100.

The patient’s information includes the name of the patient, age, sex, card number. Prescriber’s identity includes name, qualification, and signature. Drug information includes generic name prescription, dose (strength), frequency, route, quantity and/or duration and unabbreviated drug names and/or dose units. If the prescription contains more than one drug, the score was given separately for the different drugs. Drugs having their dose, frequency, route, quantity and/or duration mentioned were scored as **1**. Drug name and/or dose units that were written in abbreviated forms were scored as **0**.

It is essential to write drugs with its generic name, dose, frequency, and route of administration whenever necessary, and the drug’s name or dose units must not be abbreviated. Nonetheless, there are some approved abbreviations that may be used: g for gram, mg for milligram, microgram and nanogram should be written in full form. Doses less than 1 g should be written in milligram, less than 1 mg should be written in microgram, ml is accepted for milliliter [4].

### Assessment procedure for legibility

Measuring the legibility of prescriptions is difficult and its interpretation may be subjected to bias. The prescriptions were classified into Grade one (illegible prescription), Grade two (barely legible prescription which can be read upon the expertise of the pharmacists), Grade three (moderately legible where most of the items in the prescriptions are legible) and Grade four (completely legible prescription). Three pharmacists (two experienced pharmacists and one intern pharmacist) graded the legibility of a prescription based on the four point Likert scale. This scale would avoid the ambiguity of placing difficult to read prescriptions either as barely legible or completely illegible, and legible prescriptions as moderately legible or completely legible. Each pharmacist gave a score for each prescription individually, and the majority voting score was applied to the prescription. The decision was made by consensus when majority voting couldn’t be reached.

### Data processing and statistical analysis

The collected data were double entered on CSPro Software (version 7.0) and exported to IBM SPSS® (Version 22) for statistical analysis. Frequencies and percentages were used to summarize categorical variables. Correlates of legibility were identified using Spearman rank correlation (r_s_). Furthermore, predictors of legibility were assessed using multinomial logistic regression. Odds ratio, crude and adjusted, with 95% confidence interval was reported in bivariate and multivariable logistic regression analyses respectively. Graphs and tables were used to present the data as appropriate. All analyses were considered statistically significant when *p* < 0.05.

## Results

**Fig. 1:**
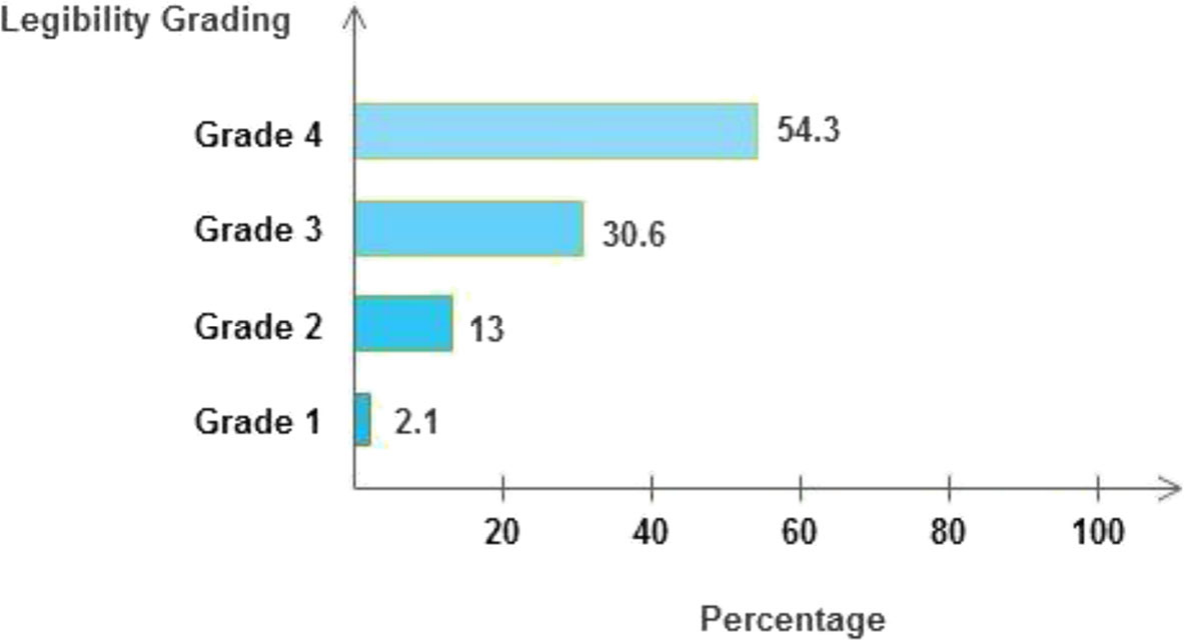
Legibility grading of the prescriptions

A total of 385 prescriptions were analyzed on which 710 drugs were prescribed. On average, a randomly selected prescription had 78.63% completeness. The patient’s age and sex were written in majority of the prescriptions and patient’s name was written in almost all of the prescriptions (Table 2). Prescriber’s name and signature were written in 75.3 and 81.8% of the prescriptions, respectively, whereas prescriber’s qualifications were included in about 39% of the total prescriptions (Table 3). Dose (strength) of the medication, frequency of administration, and route of administration were included in 83.7, 87.7 and 57.5% of the drugs prescribed respectively. The majority (83.3%) of the prescribed drugs were written in generic names (Table 4). Regarding legibility grades of the prescriptions, 54.3% of the prescriptions were in grade four, 30.6% in grade three, 13% were grade two and 2.1% were grade one (Fig. 1).

**Table 2:** Patient’s information (*N* = 385)

Variable	Completeness	
	Frequency (n)	Percentage (%)
Name of the patient	384	99.7
Age	321	83.4
Sex	345	89.6
Card number	116	30.1

**Table 3:** Prescriber information (*N* = 385)

Variable	Completeness	
	Frequency (n)	Percentage (%)
Name of the prescriber	290	75.3
Qualification	150	39
Signature	315	81.8

**Table 4:** Percentage distribution of completeness on drug information (*N* = 710)

Variable	Completeness	
	Frequency (n)	Percentage (%)
Dose	595	83.7
Frequency	623	87.7
Route	408	57.5
Quantity and/or duration*	675	95.1
Unabbreviated drug names and/or dose units**	631	88.9

Quantity and/or duration*Drugs for which either quantity or duration or both of them were written Unabbreviated drug names and dose units** Drugs having their name or dose units or both of them written

A bivariate analysis showed patient’s characteristics such as age (*p* = 0.153) and sex (*p* = 0.408) were not found to be significantly associated with legibility grading of prescriptions. Thus illegible prescriptions affected all age groups of different sex equally. A significant positive correlation was found between percentage completeness and legibility (r_s_ = 0.14, *p* = 0.006). On the other hand, a negative correlation was observed between legibility and a total number of drugs (r_s_ = − 0.226, *p* < 0.006), and the number of drugs with a brand name (not written in a generic name) (r_s_ = − 0.193, *p* < 0.001) (Table 5).

**Table 5:** Correlates of legibility (N = 385)

Variables	Legibility	
	Spearman Rank Correlation (r_**s**_)	***p***-value
Percentage completeness	0.14	0.006
Total number of drugs prescribed	−0.226	< 0.001
Number of drugs prescribed with brand name*	−0.193	< 0.001

Analysis of factors affecting legibility was performed using multinomial logistic regression for each independent variable. Generally, legibility was significantly associated with percentage completeness, a total number of drugs prescribed, and number of drugs written in a brand name (Table 6). At a multivariate level, the result showed that increasing in percentage completeness of prescriptions (AOR: 0.91, 95%CI: 0.86, 0.96) resulted in a significant reduction of encountering of illegible prescriptions (Grade one) and conversely increasing the number of prescribed drugs (AOR: 2.67, 95%CI: 1.37, 5.21) showed a significant increase of illegible prescriptions. Moreover, partially legible (Grade two) prescriptions were associated with increased number of prescribed drugs (AOR = 1.93, 95%CI: 1.36, 2.73) and number of drugs written in brand names (AOR = 1.76, 95% CI: 1.01, 3.06).

**Table 6:** Predictors of legibility using bivariate and multivariable models.

Legibility	Variables	Bivariate analysis		Multivariable analysis	
		COR (95% CI)	***p***-value	AOR (95% CI)	***p***-value
Grade one	Percentage completeness	0.92 (0.87, 0.97)	0.001	0.91 (0.86, 0.96)	**0.001**
	Total number of drugs prescribed	2.7 (1.42, 5.13)	0.003	2.67(1.37, 5.21)	**0.004**
	Number of drugs prescribed with brand name	2.44 (0.83, 7.15)	0.105	1.50(0.43, 5.20)	0.525
Grade two	Percentage completeness	0.98 (0.95, 1.00)	0.120	0.98 (0.95, 1.01)	0.203
	Total number of drugs prescribed	2.11 (1.51, 2.94)	<0.001	1.93 (1.36, 2.73)	**<0.001**
	Number of drugs prescribed with brand name	2.44 (1.44, 4.12)	0.001	1.76 (1.01, 3.06)	**0.047**
Grade three	Percentage completeness	0.98 (0.96, 1.00)	0.039	0.98 (0.96, 1.00)	0.061
	Total number of drugs prescribed	1.33 (1.01, 1.74)	0.04	1.27 (0.96, 1.69)	0.091
	Number of drugs prescribed with brand name	1.64 (1.05, 2.55)	0.029	1.43 (0.90, 2.27)	0.126

Reference Category = Grade four, *COR* Crude Odds Ratio, *AOR* Adjusted odds Ratio

## Discussion

This study focused on the completeness and legibility of hand written prescriptions and the factors associated with it. The results showed that patient’s age and sex were present in 83.4 and 89.6% of the prescriptions respectively. This finding was higher than the 67.3% reported in Malaysia [15] and 28% in Pakistan [19]. A study in Sudan [20] reported that 100% of the prescriptions didn’t fill patient’s sex. The presence of a patient’s age in a prescription, especially, in pediatric and geriatric patients is an important determinant that helps in the selection of the correct doses of a drug to the patients. Our results show that patient’s name and date of prescription were present in almost all of the prescriptions. This is similar to a study conducted in Malaysia which reported that patient’s name was present in all prescriptions [15] but higher than in India where only 50% of prescriptions had patient’s name [1]. Date of prescription in this study was comparable with other reports in Malaysia (82.9%) [15] and Pakistan (89%) [19], but they were higher than in India where only 35% of the prescriptions had dates [1].

The dose (strength), frequency and route of a drug were filled in 83.8, 87.7 and 57.5% of the prescriptions respectively. In a study done in outpatient, primary care clinic and surgery outpatient departments of a Saudi hospital, dose (strength) was missing in 8, 11.94 and 12.5% and route of medication administration was also missing in 11.11, 22.38 and 6.25% of prescriptions of the three departments, respectively [14]. Moreover, the frequency and duration of medication were not written in less than 5% [14]. Similarly, in another study, the strength of a medication, frequency of administration and route of administration were not written in 8.23, 8.87 and 13.3% respectively [21]. In Sudan, it was reported that dose and frequency were missing in 54.4 and 21.26% respectively [20]. Prescriber’s often lean towards writing the dose of the medication as one tab, two tabs or capsule instead of writing the specific dose thus leaving out frequency. In addition, some prescribers prefer to write the dosage form instead of writing the route of administration of the drug. But, the route of administration should be specified as the dosage form does not always indicate the route of administration.

In the current study, 83.3% of the drugs were written using their generic name. When compared with other studies, it was much higher than 23.3% reported in Pakistan [19], 52.41% in Sudan [20] and 56.7% in India [22]. Unless they have a valid reason to do otherwise, prescribers should always use generic names when prescribing because generic drugs are safe, accessible and offer an affordable substitute to the highly expensive branded drugs. Duration of treatment and/or quantity to be dispensed was found in 95.1% of the prescriptions (Table 4). In a similar study, duration or quantities to be dispensed were 91.2% [15]. In different studies, duration was missing in 2.02% [14] and 46.3% [19]. In another study, 27.8% of the prescriptions included the quantities of the drug to be dispensed to the patient [3].

Spearman correlation revealed that as percentage completeness of prescriptions increases, their legibility significantly increases (r_s_ = 0.14, *p* = 0.006). However, the legibility of the prescriptions decreased significantly when the total number of drugs prescribed increased (r_s_ = − 0.226, *p* < 0.006). Prescribers should, therefore, need to spend extra time to write clear and complete prescriptions to prevent any occurrence of prescription errors. As the number of non-generic drugs (brand name) in a prescription increases, its legibility was found to be decreasing (r_s_ = − 0.193, *p* < 0.001). This might be due to the prescriber’s preference to use brand names (and some of them are obsolete) which are generally difficult to interpret for the dispenser.

Due to the cross-sectional nature of the study design, the findings presented here might not reflect a cause-effect relationship. Besides, the findings of the study were based in Asmara, the capital city of Eritrea, so findings might not be generalizable to the whole country. Further nationwide research with larger sample sizes and longer duration of study should be done to measure the completeness and legibility of prescriptions in the country.

## Conclusion and recommendation

Generally, the majority of the prescriptions were written legibly with complete information. The predictor variables considered in this study are not exhaustive, future studies should include how the patient therapy related factors such as drug category; drug duplications, appropriate dose, and appropriateness of therapy affect the legibility and completeness of prescriptions. To lower prescription writing errors, prescribers should write in compliance with the national prescription guidelines, the concerned bodies should strengthen the existing laws and implementing regular awareness-raising programs are recommended.

## Supplementary information

**Additional file 1.** Data Recording Checklist Form

## Data Availability

The data used in this study is available from the corresponding author and can be accessed upon reasonable request.

## Abbreviations

AOR: Adjusted Odds Ratio
CI: Confidence Interval
COR: Crude Odds Ratio
CSPro: Census and Survey Processing System
ISMP: Institute for Safe Medication Practices
SPSS: Statistical Package for Social Sciences
WHO: World Health Organization
