# Supplementary material for: Assessment of completeness and legibility of handwritten prescriptions in six community chain pharmacies of Asmara, Eritrea: a cross-sectional study
Source: BMC Health Serv Res. 2020 Jun 22;20:570. doi: 10.1186/s12913-020-05418-9 (PMC7310160; doi:10.1186/s12913-020-05418-9)
Supplement: Supplementary file 1 — Additional file 1. Data Recording Checklist Form [file 12913_2020_5418_MOESM1_ESM.pdf]

Data Recording checklist form

No.	Indicators	Coding category
101	Prescription code	
**Section 1: Patient's Details**		
102	Name	Yes……….. 1<br>No………… 2
103	Age	Yes…………1 ⟶ Write the age __________<br>No………… 2
104	Sex	Yes…………1 ⟶ Male…… 1 Female …... 2<br>No………… 2
105	Card No	Yes……….. 1<br>No………… 2
**Section 2: The date of the prescription**		
201	Date	Yes……….. 1<br>No………… 2
**Section 3: Prescriber's Identity**		
301	Name	Yes……….. 1<br>No………… 2
302	Qualification	Yes……….. 1<br>No………… 2
303	Signature	Yes……….. 1<br>No………… 2
**Section 4: Prescribed drug name**		
401	Prescribed in a generic name	Yes……….. 1<br>No………… 2
**Section 5: Drug information**		
501	Dose	Yes……….. 1<br>No………… 2
502	Frequency	Yes……….. 1<br>No………… 2
503	Route	Yes……….. 1<br>No………… 2
504	Quantity and/or duration	Yes……….. 1<br>No………… 2
505	Abbreviation	Yes……….. 1<br>No………… 2
**Section 6: Legibility Grading**		
601	Legibility score	Grade One ……. 1<br>Grade Two……... 2<br>Grade Three……. 3<br>Grade Four……. . 4
